# Effect of alcohol consumption on oncological treatment effectiveness and toxicity in patients with cancer: a systematic review and meta-analysis

**DOI:** 10.1186/s12885-025-13694-z

**Published:** 2025-02-12

**Authors:** Georgios Fountoukidis, Aglaia Schiza, Daniel Smith, Mukhrizah Othman, Marie Bergman, Johan Ahlgren, Mats Lambe, Sandra Irenaeus, Antonis Valachis

**Affiliations:** 1 Department of Oncology, Faculty of Medicine and Health, Örebro University, Örebro, 702 81 Sweden; 2 Department of Oncology, Uppsala University Hospital, Uppsala, 751 85 Sweden; 3 Department of Immunology, Genetics and Pathology (IGP), Uppsala University, Uppsala, Sweden; 4 Clinical Epidemiology and Biostatistics, School of Medical Sciences, Örebro University, Örebro, 702 81 Sweden; 5 Department of Oncology, Hospital of Karlstad, Karlstad, 652 30 Sweden; 6 Regional Cancer Centre, Mid-Sweden Health Care Region, Uppsala, 751 22 Sweden; 7 Department of Medical Epidemiology and Biostatistics, Karolinska Institute, Stockholm, 104 35 Sweden

**Keywords:** Alcohol consumption, Cancer treatment, Effectiveness, Toxicity, Meta-analysis

## Abstract

**Background:**

Alcohol consumption has been associated with an increased risk of cancer-related mortality. It may also negatively impact oncological therapies, potentially leading to impaired effectiveness or an increased risk of treatment-related toxicities. The aim of this systematic review and meta-analysis was to examine the current evidence regarding the potential effects of alcohol consumption during cancer treatments on both treatment effectiveness and toxicity, irrespective of cancer type.

**Methods:**

A comprehensive literature search was performed across three electronic databases (Medline, Web of Science, Cochrane) covering studies from January 1990 to December 2023. Furthermore, a manual search based on the reference lists of the eligible studies was performed to identify additional potentially eligible studies. Studies were eligible if they involved cancer patients and provided data on alcohol consumption during specific oncological treatments, including its effect on treatment outcomes, or compared treatment effectiveness or toxicity between drinkers and non-drinkers. Studies were excluded if they did not meet these criteria, were duplicates, case reports, conference abstracts, or focused only on cancer-specific or overall survival.

Only studies using multivariable analyses to examine the association between alcohol consumption and treatment effectiveness or toxicity were included in the pooled analyses. Pooled Hazard Ratios (HRs) or Odds Ratios (ORs) and their corresponding 95% Confidence Intervals (CIs) were calculated using random-effects models. Study quality was assessed by using the Newcastle–Ottawa scale whereas the GRADE approach was applied to rate the certainty of evidence for pooled analyses.

**Results:**

Out of 6734 studies identified through searching, 38 met the inclusion criteria for pooled analyses. Alcohol consumption during radiotherapy, with or without concomitant chemotherapy, was associated with worse disease-free survival (pooled HR: 2.05; 95% CI: 1.09 – 3.89), although the numerically increased risk for locoregional recurrence did not reach statistically significance (pooled HR: 2.01; 95% CI: 0.76 – 5.36). The potential impact of alcohol consumption on chemotherapy-induced neurotoxicity and acute / delayed nausea was not statistically significant. However, alcohol consumption was associated with a lower risk of overall chemotherapy-induced nausea (OR: 0.69; 95% CI: 0.57, 0.84).

**Conclusion:**

Our findings suggest that alcohol consumption may have a negative impact on radiotherapy, whereas its potential impact on the effectiveness of systemic oncological therapies (chemotherapy, molecular targeted therapy, immunotherapy, endocrine therapy) has not been adequately studied. Similarly, the current evidence on the potential association between alcohol consumption and treatment-related toxicities is weak, highlighting the need for well-designed prospective studies on this topic.

**Supplementary Information:**

The online version contains supplementary material available at 10.1186/s12885-025-13694-z.

## Introduction

Alcohol consumption is associated with an increased risk of developing cancer in various organs, particularly in the head & neck region, upper gastrointestinal tract, liver, pancreas, colorectal, and breast cancer [1, 2]. Approximately 4% of all new cancer cases globally can be attributed to alcohol consumption [3]. Although the specific mechanisms may vary depending on cancer type, chronic alcohol consumption exerts a multifaceted negative impact on cancer risk through the formation of carcinogenic metabolites, promoting DNA damage, oxidative stress, inflammation, and immune suppression [4, 5]. Since alcohol consumption is a modifiable risk factor, it is considered one of the most important targets for primary cancer prevention [6, 7].

Alcohol consumption has been associated with an increased risk of cancer-related mortality for several cancer types including head-neck [8, 9] and esophageal cancer [10]. However, this association appears to be less evident and potentially without clinical significance in breast [11] and colorectal cancer [12]. Additionally, alcohol consumption is associated with an increased risk of postoperative complications and prolonged hospital stays [13, 14]. Alcohol consumption is consistently linked to increased cancer mortality and identified as a risk factor for secondary malignancies [15, 16]. Furthermore, heavy drinking and binge drinking are linked to a heightened risk of mortality from all causes, including cancer and accidents [17, 18].

The potential negative impact of alcohol consumption on oncological therapies, including impaired effectiveness or an increased risk of treatment-related toxicities, cannot be ruled out regardless of cancer type. A deeper understanding of how alcohol consumption impacts treatment efficacy, drug metabolism, and toxicity in cancer patients is essential. Adverse interactions between alcohol and certain supportive care medications can significantly on patient safety. For instance, alcohol may influence the metabolism of medications such as painkillers, antiemetics, or sedatives, resulting in reduced efficacy or increased toxicity [19–21]. While these interactions are well-documented, the effects of alcohol on chemotherapy, immunotherapy, and targeted therapies are less well understood.

Addressing these gaps could refine treatment strategies, support informed medical decisions, and enhance survivorship by elucidating the effects of alcohol on therapeutic response, disease progression, and long-term cancer outcomes.

The goal of this systematic review and meta-analysis was to consolidate the existing evidence on whether alcohol consumption might affect the effectiveness and toxicity risks in cancer patients undergoing local or systemic oncological therapeutic strategies.

## Materials and methods

This systematic review and meta-analysis protocol was prospectively registered in the PROSPERO database (CRD42022360548) and was carried out following PRISMA guidelines [22].

### Study selection

Three electronic databases—Medline, Web of Science, and Cochrane—were searched, applying the following criteria: studies published in English from January 1990 to December 2023; to ensure the inclusion of relevant, high-quality research.

In addition, we manually reviewed the reference lists of eligible studies, as well as relevant systematic reviews and meta-analyses, to identify additional studies that met our criteria.

The electronic search was conducted using a combination of keywords and MeSH terms, employing various search algorithms with Boolean operators. The search terms included alcohol intake, alcohol consumption, alcohol drinking, alcohol abuse, ethanol, cancer, chemotherapy, radiotherapy, molecular targeted therapy, immunotherapy, and endocrine therapy."

### Inclusion and exclusion criteria

#### Inclusion criteria

A study was considered eligible for inclusion if it satisfied all of the following criteria:

1. If the study included cancer patients of any tumor type.
2. If the study presented data on alcohol consumption during a specific oncological therapeutic modality regardless of the treatment method (external radiotherapy or brachytherapy, chemotherapy, targeted therapies, immunotherapy, endocrine therapy).
3. If the study provided data on the relationship between alcohol consumption and treatment outcomes, including: pathologic complete response (pCR), objective response rate (ORR), locoregional recurrence (LRR), disease-free survival (DFS), progression-free survival (PFS), and treatment-related toxicity.
4. If the study reported data on the comparison of treatment effectiveness or treatment-related toxicity between drinkers vs. non-drinkers or among patients with different amount of alcohol consumption where one category was classified as high consumption.

We included both observational and experimental studies, as long as they provided relevant information.

#### Exclusion criteria

We excluded studies if they: (1) were duplicate publications; (2) did not capture alcohol consumption during cancer treatment; (3); provided insufficient details on alcohol consumption; (4) did not present separate results on specific therapeutic oncological strategies; (5) evaluated only cancer-specific or overall survival; (6) did not analyze the relationship between alcohol consumption and treatment effectiveness or toxicity using multivariate analyses to reduce the risk of confounding bias; (7) were case reports or presented only as conference abstracts.

### Selection process

Two researchers (GF, AS) independently screened all potentially eligible studies based on their titles and abstracts. Full-text versions of these studies were then reviewed for a final eligibility assessment. The final selection of eligible studies was determined through discussions and consensus among the two initial researchers (GF, AS) and a third researcher (AV).

### Data collection process

The two researchers (GF, AS) independently gathered relevant data from each eligible study using a pre-defined form. The collected data included:

1st author’s name, publication journal, year of publication, study origin, type of study, enrollment period, number of included patients, follow-up time, cancer type, definition of alcohol consumption, treatment strategy, outcome of interest based on alcohol consumption (Odds Ratios (ORs) and corresponding 95% Confidence Intervals (CIs) for categorical outcomes; Hazard Ratios (HRs) and corresponding 95% CI for time-to-event outcomes).

### Effect measures

For alcohol consumption during radiotherapy or chemoradiotherapy (= concurrent administration of cytotoxic chemotherapy and radiotherapy), the relevant endpoints for this systematic review and meta-analysis included: LRR, DFS, and radiation-induced toxicity.

For alcohol consumption during systemic cancer treatment, the pertinent endpoints were: pCR, ORR, DFS, PFS, and treatment-related toxicity.

In terms of treatment-related toxicity, we considered all toxicities potentially linked to a specific oncological therapy as relevant. We analyzed only treatment-related toxicities that were classified based on any internationally acceptable toxicity scale as Common Terminology Criteria for Adverse Events (CTCAE any version) or Radiation Toxicity Grading (RTOG) and found to be at least grade 2 [23, 24].

### Study risk of bias assessment

The Newcastle–Ottawa scale (NOS) for cohort studies was applied by two researcher (IM, AV) for risk of bias assessment of eligible studies [25].

### Data synthesis

The hazard ratios (HR) and odds ratios (OR) from primary studies comparing alcohol consumption with non or low alcohol consumption were extracted. To standardize these comparisons, we inverted the effect sizes by taking their reciprocals, making non or low alcohol consumption the reference category across all studies. We calculated pooled effects for predefined combinations of outcomes and treatment strategies, provided there were estimates from at least three studies. The logarithms of HR and OR, along with their standard errors, were used to weight the effect sizes through the inverse variance method.

For studies reporting multiple effect sizes, a sandwich-type estimator was employed to construct a cluster-robust variance–covariance matrix, which included an adjustment for small sample sizes. Random-effects models were employed to compute pooled hazard ratios (HR) for time-to-event outcomes (LRR, DFS, PFS) and odds ratios (OR) for toxicity. The use of random-effects models was based on the nature of the statistical inference rather than on the assessment of statistical heterogeneity [26]. Statistical heterogeneity for each pooled analysis was assessed using Cochran’s Q test, with results presented as *p*-value. Publication bias for pooled analyses with a sufficient number of studies was evaluated through visual inspection of funnel plots. All statistical analyses were conducted in R (Version 4.1.0) using RStudio (Version 1.4.1717), with extensive use of the ‘metafor’ package.

### Certainty assessment

For rating the certainty of evidence regarding pooled results, the Grading of Recommendations, Assessment, Development, and Evaluation (GRADE) approach was utilized [27].

## Results

### Study selection

**Fig. 1:**
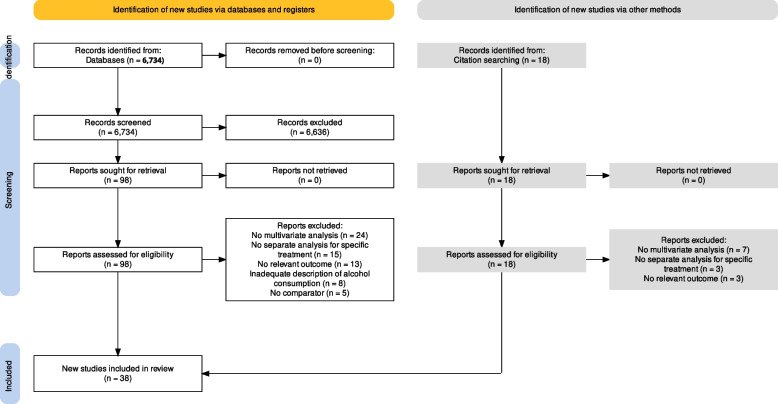
Flowchart diagram of study selection process

The electronic searching identified a total of 6734 studies to be scrutinized. After initial assessment, 98 potentially eligible studies were selected. In addition, 18 studies were selected from manual searching of relevant systematic reviews. After applying all inclusion and exclusion criteria in potentially eligible studies, 38 studies were considered eligible to be included in the systematic review and meta-analysis (Fig. 1).

To ensure the clinical relevance of the pooled analyses, data from eligible studies were pooled only if at least three studies presented results for a specific treatment strategy and a specific outcome. Seven studies did not meet the abovementioned criteria and were included only in the systematic review but not in pooled analyses. Da Silva et al. demonstrated that alcohol consumption was associated with poorer event-free survival in patients with triple-negative breast cancer. Furrer et al. found that pre-diagnosis alcohol consumption improved disease-free survival in HER2-positive breast cancer patients, while Lemieux et al. reported that high alcohol intake during trastuzumab treatment was linked to increased cardiac toxicity. Morganti et al. observed that wine consumption was associated with reduced acute skin toxicity in breast cancer patients undergoing radiotherapy. Raguse et al. identified alcohol as a significant univariate risk factor for osteoradionecrosis in head and neck cancer patients. Reibolt et al. showed that high alcohol consumption increased cardiotoxicity risk in breast cancer patients, whereas moderate consumption reduced the risk. Stankovic et al. suggested that alcohol consumption might be a potential predictive factor for acute gastrointestinal toxicity in prostate cancer patients receiving radiotherapy. (Supplementary, narrative summary and references).

### Characteristics of eligible studies

The majority of eligible studies (26 of 38; 68%) were retrospective cohort studies, followed by prospective cohort studies (10 of 38; 26%), and two (5%) were randomized controlled trial. The studies covered a wide range of cancer types, with head & neck malignancies being the most common (11 of 38; 29%), followed by breast cancer (10 of 38; 26%). The number of patients included ranged from 69 to 1923 patients.

A detailed description of eligible studies is presented in Supplementary Table 1, while the overall quality assessment is shown in Supplementary Table 2.

Based on the available evidence, three specific clinical situations were identified for which pooled analyses were able to be performed: effectiveness of radiotherapy with or without concomitant chemotherapy in drinkers compared to non-drinkers, risk for chemotherapy-induced neurotoxicity, and risk for chemotherapy-induced nausea.

### Impact of alcohol consumption on radiotherapy +/- concomitant chemotherapy

The impact of alcohol consumption during radiotherapy was analyzed for two outcomes of interest, namely LRR and DFS.

**Fig. 2:**
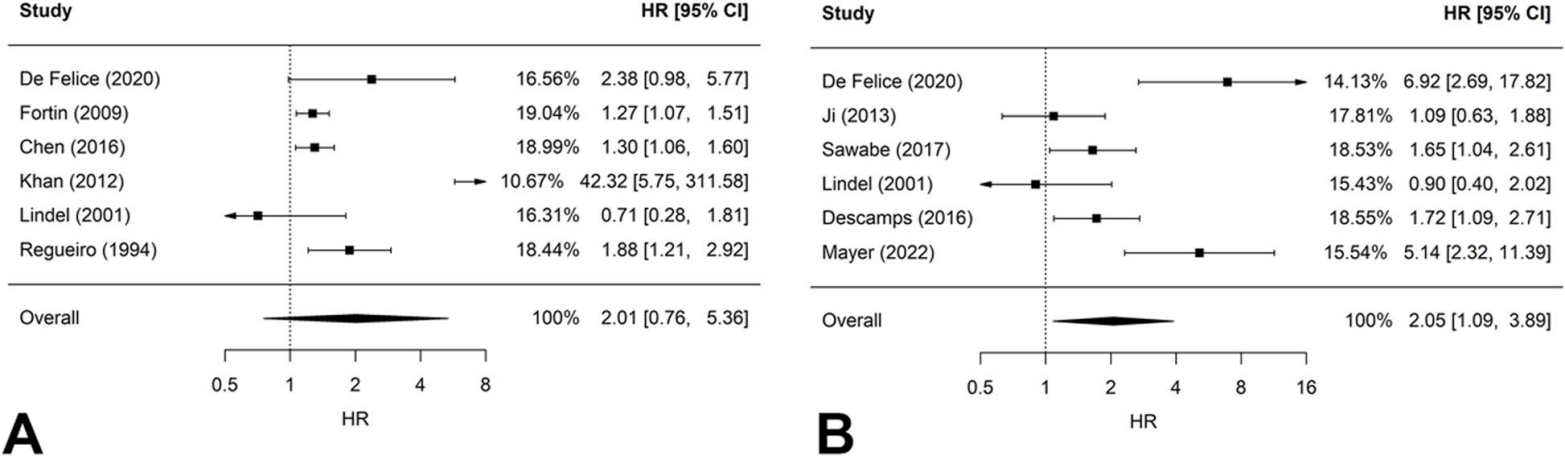
Forest plots on alcohol consumption during radiotherapy +/- concomitant chemotherapy and **A** locoregional recurrence; **B** disease-free survival. HR > 1 indicates worse outcome for patients with alcohol consumption during radiotherapy whereas HR < 1 indicates better outcome. The comparison group consists of patients without any alcohol consumption except from Regueiro et al. where the comparison group consists of patients with low alcohol consumption

Six studies (*N* = 3870) presented primary results on the association between alcohol consumption during radiotherapy +/- concomitant chemotherapy and LRR. The pooled HR for LRR was 2.01 with 95% CI 0.76–5.36 (*P*-value for heterogeneity = 0.003; Fig. 2A) To assess the robustness of the findings, a sensitivity analysis was conducted after excluding one study that included patients with low alcohol consumption in the comparison group. The sensitivity analysis was similar to primary analysis (pooled HR: 2.14: 95% CI: 0.61 – 7.54; *P*-value for heterogeneity = 0.004).

Six studies, involving a total of 1242 patients, evaluated the impact of alcohol consumption on DFS in patients undergoing radiotherapy +/- concomitant chemotherapy. The pooled HR for DFS was 2.05 (95% CI: 1.09–3.89; *P*-value for heterogeneity < 0.001; Fig. 2B).

### Alcohol consumption and chemotherapy-induced neurotoxicity

**Fig. 3:**
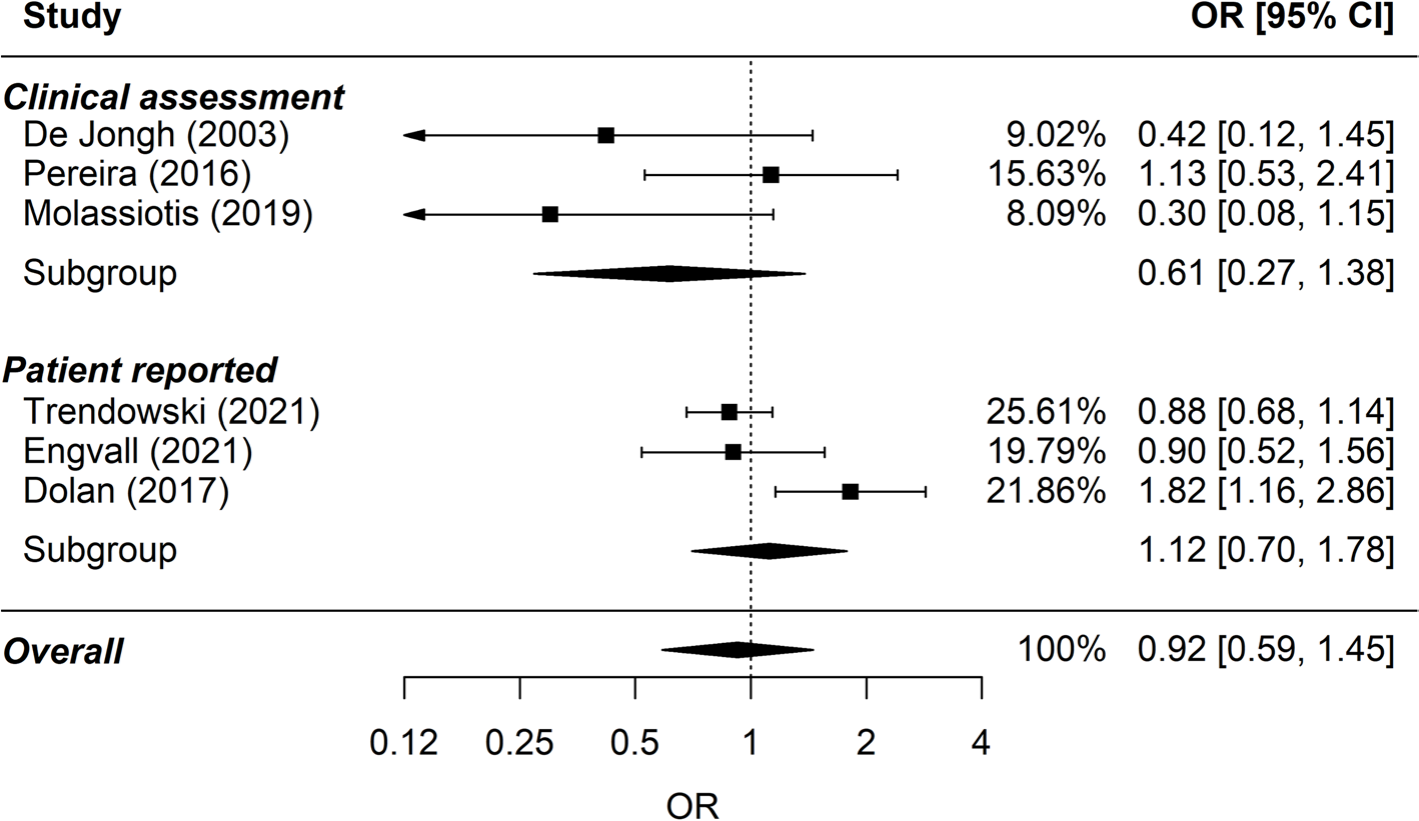
Forest plot on alcohol consumption and chemotherapy-induced neurotoxicity. OR > 1 indicates higher risk in drinkers. The comparison group consists of patients without any alcohol consumption except from Dolan et al. where the comparison group consisted of patients with low alcohol consumption

No association was found between alcohol consumption and chemotherapy-induced neurotoxicity in patients treated with platinum-based and/or taxane-based chemotherapy (6 studies; 3109 patients) (pooled OR: 0.92; 95% CI: 0.59–1.45; *P*-value for heterogeneity = 0.024; Fig. 3). The pooled OR remained non-significant in the sensitivity analysis where one study included patients with low alcohol consumption as the reference was excluded (pooled OR: 0.84; 95% CI: 0.64 – 1.11; *P*-value for heterogeneity = 0.380).

The toxicity assessment strategies (patient-reported or clinical assessment) did not impact the results.

### Alcohol consumption and chemotherapy-induced nausea

The potential association between alcohol consumption and chemotherapy-induced nausea was evaluated in various aspects (acute, delayed, overall nausea) and assessment strategies (patient-reported, clinical assessment, not specified).

**Fig. 4:**
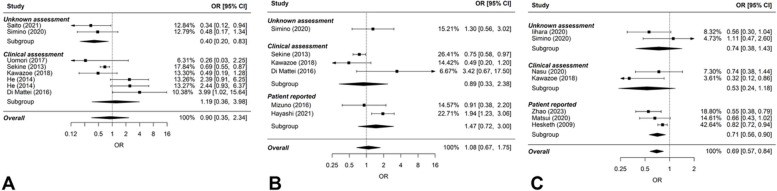
Forest plots on alcohol consumption and chemotherapy-induced **A** acute nausea; **B** delayed nausea; **C** any nausea. OR > 1 indicates higher risk in drinkers. The comparison group consists of non-drinkers

Alcohol consumption was not associated with either acute or delayed nausea regardless of the assessment strategy used (Fig. 4A, B). However, a lower risk for overall nausea was found (7 studies; 3345 patients; pooled OR: 0.69; 95% CI: 0.57 – 0.84; *P*-value for heterogeneity = 0.150; Fig. 4C). The latter seemed to be driven mainly by studies that used patient-reported outcomes to capture nausea.

### Publication bias of pooled analyses

The funnel plots investigating publication bias of the main pooled analyses are presented as Supplementary Figures (Supplementary Figs. 1—6). Although their interpretation should be done with caution due to the limited number of studies in the pooled analyses, publication bias cannot be ruled out. This is particularly true for analyses related to alcohol consumption during radiotherapy +/- concomitant chemotherapy, as well as the impact of alcohol on LRR, DFS, and overall nausea.

### Certainty of evidence based on GRADE

The certainty of evidence for each pooled analysis using the GRADE approach, is displayed in Table 1. The level of certainty ranged from very low to low. Specifically, for the two pooled analyses with statistically significant results, the negative effect of alcohol consumption on DFS in patients receiving radiotherapy +/- chemotherapy was graded as low whereas the certainty of evidence on the association between alcohol consumption and chemotherapy-induced nausea was very low. This is attributed to the presence of inconsistent results among eligible studies as well as the utilization of different methods to measure nausea.

**Table 1:** Certainty of evidence according to GRADE approach for pooled analyses investigating the impact of alcohol consumption on treatment effectiveness or treatment-related toxicity

N studies	Assessment of evidence						Effect		Certainty of evidence
	**Study design**	**Bias**	**Inconsistency**	**Indirectness**	**Imprecision**	**Other**	**N patients**	**Pooled effect (95% CI)**	
Locoregional recurrence after radiotherapy (+/- chemotherapy)									
6	observation	Serious^a^	Serious^b^	Not serious	Not serious	No	3870	2.01 (0.76–5.36)	⨁⨁◯◯ Low
Disease-free survival after radiotherapy (+/- chemotherapy)									
6	observation	Serious^a^	Serious^b^	Not serious	Not serious	No	1242	2.05 (1.09–3.89)	⨁⨁◯◯ Low
Chemotherapy-induced neurotoxicity									
6	observation	Serious^a^	Serious^c^	Not serious	Serious^d^	No	3109	0.92 (0.59–1.45)	⨁◯◯◯ Very low
Chemotherapy-induced acute nausea in patients who drink vs those who do not									
7	observation	Serious^a^	Serious^c^	Not serious	Serious^d^	No	2436	0.90 (0.35–2.34)	⨁◯◯◯ Very low
Chemotherapy-induced delayed nausea in patients who drink vs those who do not									
6	observation	Serious^a^	Serious^c^	Not serious	Serious^d^	No	2500	1.08 (0.67–1.75)	⨁◯◯◯ Very low
Chemotherapy-induced nausea (acute or delayed) in patients who drink vs those who do not									
7	observation	Serious^a^	Serious^c^	Not serious	Serious^d^	No	3345	0.69 (0.57–0.84)	⨁◯◯◯ Very low

^a^Retrospective studies with inherent risk for bias

^b^Statistical heterogeneity in pooled analyses

^c^Incosistent point estimates of eligible studies accompanied with broad confidence intervals

^d^Broad confidence interval of the pooled estimates

### Risk of bias based on Newcastle–Ottawa Scale (NOS)

NOS was applied to assess the risk of bias across the domains of selection, comparability, and outcome. The total scores for the included studies ranged from 4 to 9, reflecting varying levels of methodological quality. Five studies were categorized as high quality, scoring 9 points and eleven were deemed moderate quality, scoring between 7 and 8 points. On the other hand, twenty-one studies were assessed as lower quality, scoring between 4 and 6 points and three studies scored the lowest with 4 points.

## Discussion

The present systematic review and meta-analysis provides interesting insights and highlight knowledge gaps regarding the potential associations between alcohol consumption and the effectiveness or treatment-related toxicity of oncological therapies. Alcohol consumption during radiotherapy, with or without concomitant chemotherapy, appears to negatively impact the risk of recurrence, while there is a lack of evidence regarding the potential effect of alcohol consumption on systemic oncological therapies. A reduced risk for chemotherapy-induced nausea with alcohol consumption was noticed, although theabsence of a similar association in acute or delayed nausea and the low level of evidence precludes any firm conclusion.

The negative impact of alcohol consumption on the effectiveness of radiotherapy is supported by both preclinical and clinical evidence. Several potential mechanisms have been proposed, including an increased risk of genomic alterations caused by alcohol consumption, which can lead to treatment resistance [23, 24]. Additionally, alcohol use may deteriorate patients' nutritional status, potentially affecting treatment compliance and effectiveness [25]. Immunosuppression associated with alcohol consumption has also been suggested as a contributing factor [26].

The latter is of particular importance considering the role of immune system in cancer progression and the emerging role of checkpoint inhibitors as treatment strategy in various cancer types. Preclinical evidence has shown that alcohol consumption suppresses both the cellular and antigen-driven immunity which could theoretically impact the immunomodulatory effect of checkpoint inhibitors and thus their effectiveness [28, 29]. The potential impact of alcohol consumption on immunotherapy with checkpoint inhibitors remains an area requiring further research.

The potential positive impact of alcohol consumption on chemotherapy-induced nausea has been hypothesized to be related to a reduced sensitivity of chemoreceptor trigger zone due to the chronic exposure to alcohol [30]. Although our findings partially support this hypothesis, caution is needed when interpreting the results considering the very low certainty of evidence as well as the lack of similar association in pooled analyses of studies investigating acute or delayed chemotherapy-induced nausea.

Our findings should be interpreted in light of both the strengths and limitations of the primary studies included in the meta-analysis as well the inherent limitations of the meta-analytical approach *per se*. The adoption of wider inclusion criteria, focusing on treatment modalities rather than on specific cancer types, facilitates the generalizability of study results to a broader population of cancer patients. Furthermore, the inclusion of primary studies investigating the role of alcohol consumption in cancer therapy through multivariate analyses only was an effort to reduce the risk of confounding and selection biases, which are common in observational studies [31]. However, it is noteworthy that the risk for residual bias remains a concern even when only multivariate analyses are considered for pooled analyses, as reflected in the certainty of evidence in our findings, which ranges from very low to low. The use of the GRADE approach to rate the level of evidence is a methodological strength of the study, as it provides a structured framework for assessing evidence and enhances the interpretability of our pooled results in a clinical context. Nonetheless, it is important to note that the GRADE assessment indicated a low to very low certainty of evidence, which should be taken into consideration when applying these findings.

Our study has several limitations that should be acknowledged. The heterogeneity among eligible studies is a major concern that can impact the validity of pooled results. Multiple sources of between-study heterogeneity were observed, including differences in the definition and measurement strategies of alcohol consumption, the definition of the reference group for comparisons, and the methods for measuring relevant endpoints. To investigate the impact of between-study heterogeneity related to the definition of the reference group, we performed sensitivity analyses by including only studies that defined non-drinkers as the reference group (hence excluding studies that included patients with low alcohol consumption as the reference group) and found similar resultsto the main analyses. Furthermore, subgroup analyses based on different measurement strategies for toxicities were performed to assess the impact of these strategies on study results. Another limitation was the lack of relevant information to conduct a dose–response analysis between alcohol consumption and treatment effectiveness or toxicity that could enhance the reliability of any potential association. Further limitations include the risk of residual bias in eligible studies despite the adoption of multivariate analyses and limited or absent of evidence for systemic oncological therapies and outcomes of potential interest. Additionally, there is a risk of positive-outcome bias, where studies showing no association between alcohol consumption and outcomes might be underreported [32]. To evaluate the quality of eligible studies, we applied the NOS to each eligible observational study as a part of the GRADE approach for rating the certainty of evidence. Although NOS is a widely used tool for quality assessment of observational studies it has some limitations. The inherent subjectivity in scoring, along with the lack of validation and a uniform definition of the threshold for high quality studies, should be recognized as a potential limitation in study quality assessment.

This systematic review highlights several critical knowledge gaps that could guide future research efforts, particularly regarding the impact of alcohol consumption on the effectiveness of systemic oncological therapies as chemotherapy, targeted therapies, and immunotherapy. Additionally, it highlights the need to investigate whether alcohol consumption might affect other important outcomes, such as pathological complete response, response rates, and progression-free survival, as well as its potential influence on treatment-related toxicities beyond chemotherapy-induced nausea and neurotoxicity. Furthermore, the review has identified methodological drawbacks among the eligible studies that should be addressed in future studies. Specifically, future studies should utilize reliable and validated methods to measure and define alcohol consumption at different levels to facilitate dose–response analyses and ensure accurate measurement of outcomes to prevent misclassification and selection bias.

In summary, our findings suggest that alcohol consumption may have a negative impact on radiotherapy whereas its potential impact on the effectiveness of systemic oncological therapies has not been adequately studied. Similarly, the current evidence on the potential association between alcohol consumption and treatment-related toxicities is weak, thus highlighting the need for well-designed prospective studies in this field. This systematic review has highlighted existing knowledge gaps and proposed methodological approaches to mitigate common sources of bias, offering guidance for the design and execution of future studies.

## Supplementary Information

Supplementary Material 1.

## Data Availability

The datasets generated and analysed during the current study are not publicly available but are available from the corresponding author on reasonable request.
