# Supplementary material for: Effect of alcohol consumption on oncological treatment effectiveness and toxicity in patients with cancer: a systematic review and meta-analysis
Source: BMC Cancer. 2025 Feb 12;25:246. doi: 10.1186/s12885-025-13694-z (PMC11823036; doi:10.1186/s12885-025-13694-z)
Supplement: Supplementary file 1 — Supplementary Material 1. [file 12885_2025_13694_MOESM1_ESM.docx]

Supplementary Table 1. Characteristics of eligible studies

| **Author, year** | **Study design** | **Cancer type** | **Treatment** | **Definition of alcohol intake** | **Data source for alcohol intake** | **N patients** | **Outcome measures** | **Included in pooled analyses** |
| --- | --- | --- | --- | --- | --- | --- | --- | --- |
| Chen, 2016 | Retrospective cohort | H & N | Radiotherapy | No consumption vs. quitted > 6 mo vs. consumer | Medical records | 1923 | Locoregional recurrence | Yes |
| da Silva, 2020 | Retrospective cohort | Breast cancer (Triple Negative) | Chemotherapy (various neoadjuvant) | Any alcohol consumption | Medical records | 235 | Pathologic complete response and Event-free survival | No |
| De Felice, 2020 | Prospective cohort | H & N | Chemoradiotherapy | Alcohol abuse | Medical records | 69 | Locoregional recurrence and invasive-disease free survival | Yes |
| de Jongh, 2003 | Retrospective cohort | Various solid tumors | Chemotherapy (cisplatin) | Alcohol intake >2 units per day | Medical records | 400 | Toxicity | Yes |
| Descamps, 2016 | Retrospective cohort | H & N | Chemoradiotherapy (CRT) | never used alcohol or continued intake during treatment or stopped intake at/before diagnosis | Medical records | 217 | Disease-free survival | Yes |
| Di Mattei, 2016 | Prospective cohort | Gynaecological cancer | Radiotherapy | Any alcohol consumption | Patient-reported | 94 | Toxicity | Yes |
| Dolan, 2017 | Retrospective cohort | Germ cell tumor | Chemotherapy (cisplatin) | < 2 drinks per day or excessive drinkers (defined as those who reported consuming ≥ 2 drinks/day on average in the past year). | Patient-reported | 677 | Toxicity | Yes |
| Engvall, 2021 | Retrospective cohort | Breast cancer | Chemotherapy (taxane) | Alcohol risk consumption measured by AUDIT-C , more than 35 units/week for women | Patient-reported and medical records | 646 | Toxicity | Yes |
| Fortin, 2009 | Retrospective cohort | H & N | Radiotherapy and chemoradiotherapy | Any alcohol consumption | Medical records | 1411 | Local control | Yes |
| Furrer, 2018 | Retrospective cohort | Breast cancer | Chemotherapy (trastuzumab) | Alcohol consumption during trastuzumab treatment. Quantity of 0-2 drinks per week or > 2 drinks per week | Patient-reported | 128 | Disease free survival | No |
| Hayashi, 2021 | Prospective cohort | Colorectal cancer | Chemotherapy (oxaliplatin) | Drinking habit | Register-based | 272 | Toxicity | Yes |
| He, 2014 | Prospective cohort | Acute myeloid leukemia | Chemotherapy (cytarabine) | History of drinking | Medical records | 215 | Toxicity | Yes |
| Hesketh, 2009 | Randomised clinical trial | Various solid tumors | Chemotherapy (cisplatin) | Alcoholic drinks/week; 0-4 (light/non-drinker), ≥5 (drinker) | Patient-reported | 1043 | Toxicity | Yes |
| Iihara, 2020 | Retrospective cohort | Various cancers | Chemotherapy (carboplatin) | Habitual alcohol consumption: yes/no | Medical records | 385 | Toxicity | Yes |
| Ji, 2013 | Retrospective cohort | H & N | Radiotherapy and chemoradiotherapy | Alcohol consumption (never/ever) | Medical records | 276 | Relapse free survival (LR) and disease free survival | Yes |
| Kawazoe, 2018 | Retrospective cohort | Breast cancer | Chemotherapy (anthracycline) | History of alcohol habit (yes or no) | Medical records | 103 | Toxicity | Yes |
| Khan, 2012 | Retrospective cohort | H & N | Radiotherapy | Heavy alcohol use (never, former, current and unknown) | Medical records | 129 | Local recurrence | Yes |
| Lemieux, 2013 | Retrospective cohort | Breast cancer | Targeted therapy (trastuzumab) | Alcohol use/week during trastuzumab (0-2; 3-9; 10+) | Patient-reported | 129 | Toxicity | No |
| Lindel, 2001 | Retrospective cohort | H & N | Radiotherapy | Consumption of alcohol either in former times or at the time of recording the medical history were classified as alcohol consumers | Medical records | 99 | Local failure free survival and disease-free survival | Yes |
| Matsui, 2020 | Prospective cohort | Various solid tumors | Radiotherapy | Alcohol intake history | Medical records | 386 | Toxicity | Yes |
| Mayadev, 2017 | Retrospective cohort | Cervical cancer | Radiotherapy, brachytherapy, CRT | CDC definition of heavy alcohol consumption being >1 drinks per day on average | Medical records | 95 | Disease-free survival | Yes |
| Mayer, 2022 | Retrospective cohort | H & N | Chemoradiotherapy | Alcohol abuse (never/terminated/current) | Medical records | 154 | Disease-free survival | Yes |
| Mizuno, 2016 | Prospective cohort | Gynecological cancer | Chemotherapy (various types) | Drinking habit of five times/week or more | Medical records | 214 | Toxicity | Yes |
| Molassiotis, 2019 | Retrospective cohort | Various cancers | Chemotherapy (platinum, taxane) | Alcohol intake history | Medical records | 255 | Toxicity | Yes |
| Morganti, 2009 | Retrospective cohort | Breast cancer | Radiotherapy | Non-drinkers: alcohol intake < once a week. Drinkers: wine intake of 0.5, 1, 2, and ≥3 glasses daily | Patient-reported and medical records | 348 | Toxicity | No |
| Nasu, 2020 | Retrospective cohort | Various solid tumors | Chemotherapy (carboplatin) | History of habitual alcohol intake | Medical records | 314 | Toxicity | Yes |
| Pereira, 2016 | Prospective cohort | Breast cancer | Chemotherapy (various neoadjuvant and adjuvant) | At least one alcoholic drink per month (≤1 standard drink; >1 standard drink), and stopped drinking at least 6 months before the interview | Patient-reported and medical records | 91 | Toxicity | Yes |
| Raguse, 2016 | Retrospective cohort | H & N | Radiotherapy | No alcohol pre-RT and post-RT, and alcohol consumption pre-RT and post-RT | Medical records | 185 | Toxicity | No |
| Regueiro, 1994 | Retrospective cohort | H & N | Radiotherapy | Alcohol intake < or > 40 g per day | Medical records | 239 | Local control and disease-free survival | Yes |
| Reinbolt, 2016 | Retrospective cohort | Breast cancer | Chemotherapy (anthracycline) | < or > 8 drinks per week | Medical records | 161 | Toxicity | No |
| Saito, 2021 | Retrospective cohort | Breast cancer | Chemotherapy (anthracycline) | Alcohol intake ≥5 days in a week | Medical records | 126 | Toxicity | Yes |
| Sawabe, 2017 | Prospective cohort | H & N | Chemotherapy (platinum), radiotherapy and CRT | Alcohol consumption was converted to ethanol per day: light (<23 g ethanol/day); moderate (23–46 g ethanol/day), and heavy drinker (>46 g ethanol/day) | Patient-reported | 427 | Disease-free survival | Yes |
| Sekine, 2013 | Prospective cohort | Various cancer types | Chemotherapy (various types) | alcohol consumption (habitual versus non‐habitual) | Medical records | 1549 | Toxicity | Yes |
| Simino, 2020 | Prospective cohort | Various cancer types | Chemotherapy (various types) | Any alcohol consumption | Medical records | 268 | Toxicity | Yes |
| Stankovic, 2016 | Retrospective cohort | Prostate cancer | Radiotherapy | Regular and occasional alcohol consumption | Medical records | 81 | Toxicity | No |
| Trendowski, 2021 | Retrospective cohort | Various cancer types | Chemotherapy (not specified) | Alcohol consumption was assessed qualitatively as consuming at least one alcoholic beverage in the 4 weeks prior to survey completion and quantitatively as number of standard drinks per week | Patient-reported | 1040 | Toxicity | Yes |
| Uomori, 2017 | Retrospective cohort | Breast cancer | Chemotherapy (anthracycline) | no drinking (none or less than once a week), habitual (drinking alcohol more than three times a week), social (less than three times but at least once a week) | Patient-reported | 81 | Toxicity | Yes |
| Zhao, 2023 | Randomized controlled | Various cancer types | Chemotherapy (cisplatin) | Any alcohol consumption | Patient-reported | 706 | Toxicity | Yes |

**A narrative summary of the outcomes reported and findings from the 7 studies that were included only in the systematic review.**

Da Silva et al. [1] conducted a retrospective study involving 235 patients to investigate the relationship between sociodemographic factors and outcomes in triple-negative breast cancer patients undergoing neoadjuvant chemotherapy. Their analysis, based on medical records, revealed that any alcohol consumption was associated with worse event-free survival (adjusted HR = 1.67, p = 0.006).

Furrer et al. [2] performed a retrospective cohort study to evaluate the impact of alcohol consumption, assessed through validated questionnaires, on disease-free survival (DFS) in 128 HER2-positive breast cancer patients treated with trastuzumab. They found that pre-diagnosis alcohol consumption significantly improved DFS (HR = 0.56, p = 0.03) compared to non-drinkers. However, no association was observed between alcohol consumption during trastuzumab treatment and DFS.

Lemieux et al. [3], in a retrospective cohort study involving 237 women with non-metastatic HER2-positive breast cancer treated with trastuzumab, examined the relationship between alcohol use, assessed through questionnaires, and trastuzumab-associated cardiac toxicity. The study found that consuming 10 or more alcoholic drinks per week during treatment was linked to an increased risk of cardiac toxicity (p = 0.04).

Morganti et al. [4]analyzed 348 breast cancer patients to assess risk factors for reduced skin toxicity. Patient medical history and habits, including wine consumption, were recorded before treatment. Multivariate analysis revealed that wine intake was correlated with a reduction in acute skin toxicity.

Raguse et al. [5] conducted a retrospective cohort study to assess risk factors for osteoradionecrosis (ORN) of the jaw in 185 head and neck cancer patients undergoing radiotherapy. While alcohol consumption was a significant univariate risk factor for ORN, it did not remain significant in the multivariate analysis.

Reibolt et al. [6] performed a retrospective cohort study on breast cancer patients treated with Adriamycin and Cytoxan to identify risk factors for cardiotoxicity. Among 52 cases of cardiomyopathy and 110 controls, multivariate analysis showed that consuming more than eight alcoholic drinks per week was associated with increased cardiotoxicity risk (p= 0.024), whereas moderate alcohol consumption (<8 drinks/week) was linked to a lower risk (p= 0.009).

Stankovic et al. [7] evaluated 81 prostate cancer patients undergoing radiotherapy in a retrospective study. Univariate logistic regression analysis identified alcohol consumption (p = 0.068) as a potential predictive factor for acute gastrointestinal toxicity of any grade.

**Referenses**

1. da Silva, J.L., et al., *Sociodemographic, Clinical, and Pathological Factors Influencing Outcomes in Locally Advanced Triple Negative Breast Cancer: A Brazilian Cohort.* Breast Cancer (Auckl), 2020. **14**: p. 1178223420962488.

2. Furrer, D., et al., *Association of Tobacco Use, Alcohol Consumption and HER2 Polymorphisms With Response to Trastuzumab in HER2-Positive Breast Cancer Patients.* Clin Breast Cancer, 2018. **18**(4): p. e687-e694.

3. Lemieux, J., et al., *Alcohol and HER2 polymorphisms as risk factor for cardiotoxicity in breast cancer treated with trastuzumab.* Anticancer Res, 2013. **33**(6): p. 2569-76.

4. Morganti, A.G., et al., *Radioprotective effect of moderate wine consumption in patients with breast carcinoma.* Int J Radiat Oncol Biol Phys, 2009. **74**(5): p. 1501-5.

5. Raguse, J.D., et al., *Patient and treatment-related risk factors for osteoradionecrosis of the jaw in patients with head and neck cancer.* Oral Surg Oral Med Oral Pathol Oral Radiol, 2016. **121**(3): p. 215-21 e1.

6. Reinbolt, R.E., et al., *Risk factors for anthracycline-associated cardiotoxicity.* Support Care Cancer, 2016. **24**(5): p. 2173-2180.

7. Stankovic, V., et al., *Toxicity of the lower gastrointestinal tract and its predictive factors after 72Gy conventionally fractionated 3D conformal radiotherapy of localized prostate cancer.* J BUON, 2016. **21**(5): p. 1224-1232.

Supplementary Table 2. Quality assessment of eligible studies using the Newcastle-Ottawa scale

| **Author, year** | **Selection** | | | | **Comparability** | | **Outcome** | | | **Total** |
| --- | --- | --- | --- | --- | --- | --- | --- | --- | --- | --- |
|  | Representativeness | Selection of non-exposed | Ascertainment | No outcome at baseline | Comparable for year at diagnosis | Comparable for other factors | Assessment of outcome | Adequate follow-up  (> 3 years) | Loss to follow-up  (< 10%) |  |
| Chen, 2016 | * | * | * | * | * | * | * | * | * | 9 |
| da Silva, 2020 | * | * | * | * | * | * | * | * | * | 9 |
| De Felice, 2020 | * | * | * | * | * | * | * | * | * | 9 |
| de Jongh, 2003 | * | * | * | * | - | - | * | - | * | 6 |
| Descamps, 2016 | * | * | * | * | - | - | * | - | * | 6 |
| Di Mattei, 2016 | * | * | - | * | * | - | - | - | * | 5 |
| Dolan, 2017 | * | * | - | * | * | - | - | - | - | 4 |
| Engvall, 2021 | * | * | * | * | * | * | - | * | * | 8 |
| Fortin, 2009 | * | * | * | * | * | * | * | * | * | 9 |
| Furrer, 2018 | * | * | - | * | * | - | * | * | * | 7 |
| Hayashi, 2021 | * | * | * | * | * | * | - | - | - | 6 |
| He, 2014 | * | * | * | * | * | - | - | - | * | 6 |
| Hesketh, 2009 | * | * | - | * | * | - | - | - | - | 4 |
| Iihara, 2020 | * | * | * | * | * | - | - | - | - | 5 |
| Ji, 2013 | * | * | * | * | * | * | * | - | * | 8 |
| Kawazoe, 2018 | * | * | * | * | * | * | - | - | * | 7 |
| Khan, 2012 | * | * | * | * | - | - | * | * | * | 7 |
| Lemieux, 2013 | * | * | - | * | * | - | * | - | - | 5 |
| Lindel, 2001 | * | * | * | * | - | - | * | - | - | 5 |
| Matsui, 2020 | * | * | * | * | * | * | - | - | - | 6 |
| Mayadev, 2017 | * | * | * | * | * | * | * | - | * | 8 |
| Mayer, 2022 | * | * | * | * | * | * | * | * | * | 9 |
| Mizuno, 2016 | * | * | * | * | - | - | - | - | - | 4 |
| Molassiotis, 2019 | * | * | * | * | * | * | - | - | * | 7 |
| Morganti, 2009 | * | * | * | * | - | - | * | * | * | 7 |
| Nasu, 2020 | * | * | * | * | * | - | - | - | * | 6 |
| Pereira, 2016 | * | * | * | * | - | * | - | * | * | 7 |
| Raguse, 2016 | * | * | * | * | - | - | * | * | * | 7 |
| Regueiro, 1994 | * | * | * | * | - | - | * | - | * | 6 |
| Reinbolt, 2016 | * | * | * | * | - | - | * | - | * | 6 |
| Saito, 2021 | * | * | * | * | * | * | - | - | - | 6 |
| Sawabe, 2017 | * | * | - | * | * | * | * | * | * | 8 |
| Sekine, 2013 | * | * | * | * | * | * | - | - | - | 6 |
| Simino, 2020 | * | * | * | * | * | - | - | - | * | 6 |
| Stankovic, 2016 | * | * | * | * | - | - | * | - | * | 6 |
| Trendowski, 2021 | * | * | - | * | * | * | - | - | - | 5 |
| Uomori, 2017 | * | * | - | * | * | * | - | - | - | 5 |
| Zhao, 2023 | * | * | * | * | * | * | * | * | - | 8 |

Supplementary figures


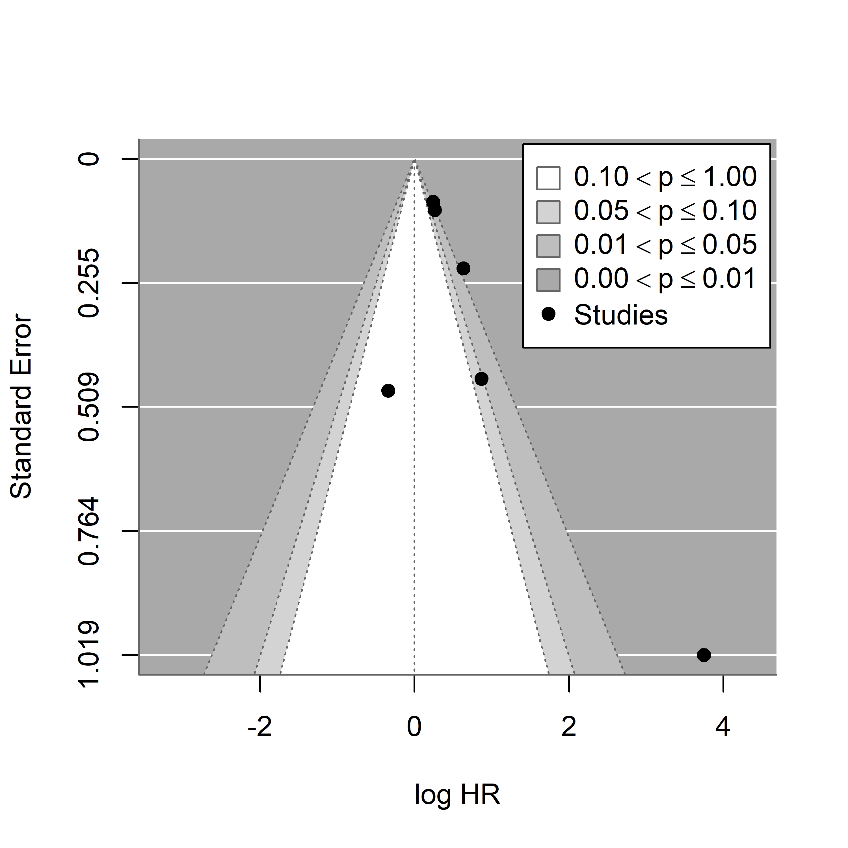


Supplementary Figure 1. Funnel plot for the pooled analysis on alcohol consumption during radiotherapy +/- concomitant chemotherapy and locoregional recurrence


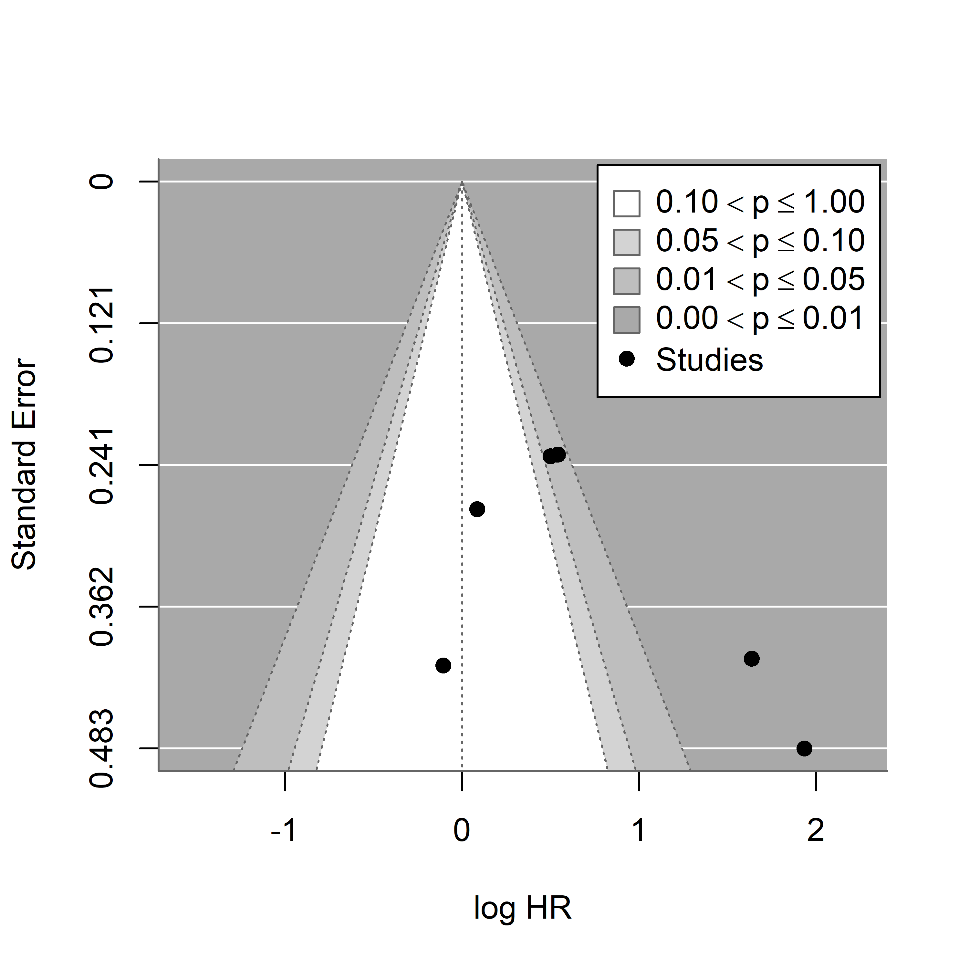


Supplementary Figure 2. Funnel plot for the pooled analysis on alcohol consumption during radiotherapy +/- concomitant chemotherapy and disease-free survival


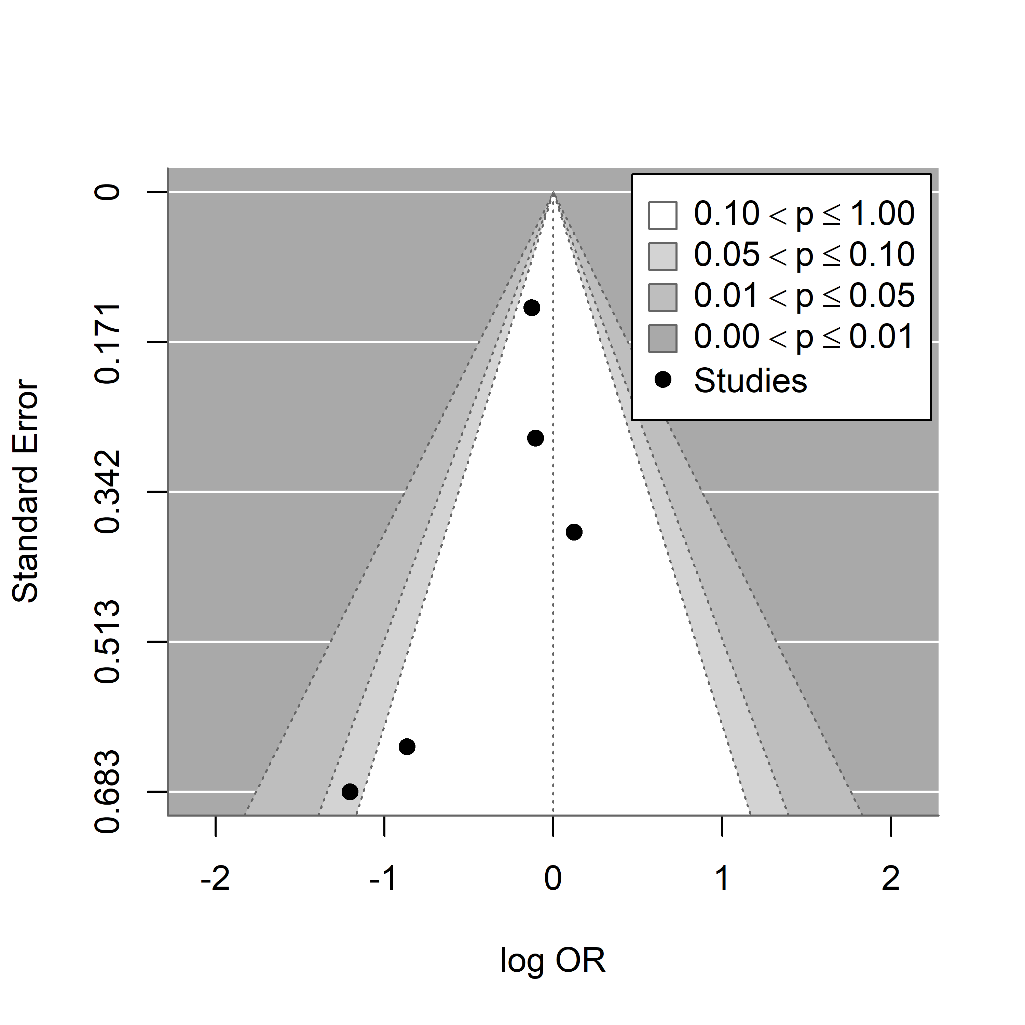


Supplementary Figure 3. Funnel plot for the pooled analysis on alcohol consumption and chemotherapy-induced neurotoxicity


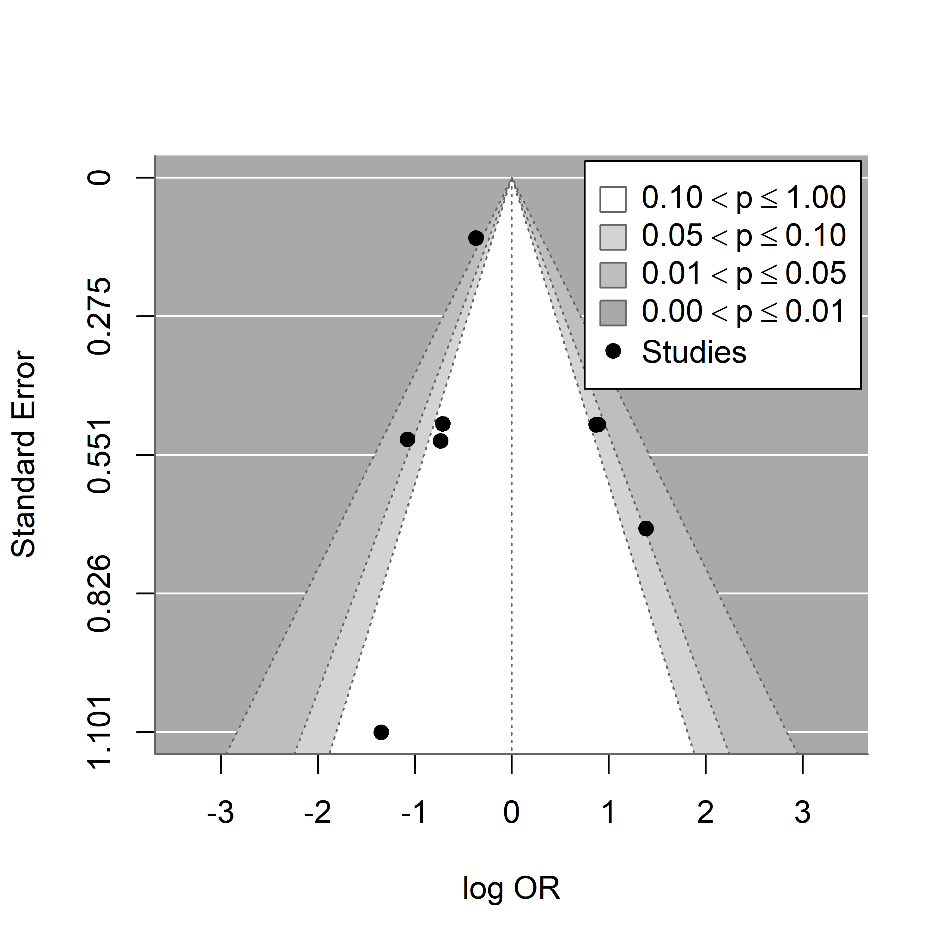


Supplementary Figure 4. Funnel plot for the pooled analysis on alcohol consumption and chemotherapy-induced acute nausea


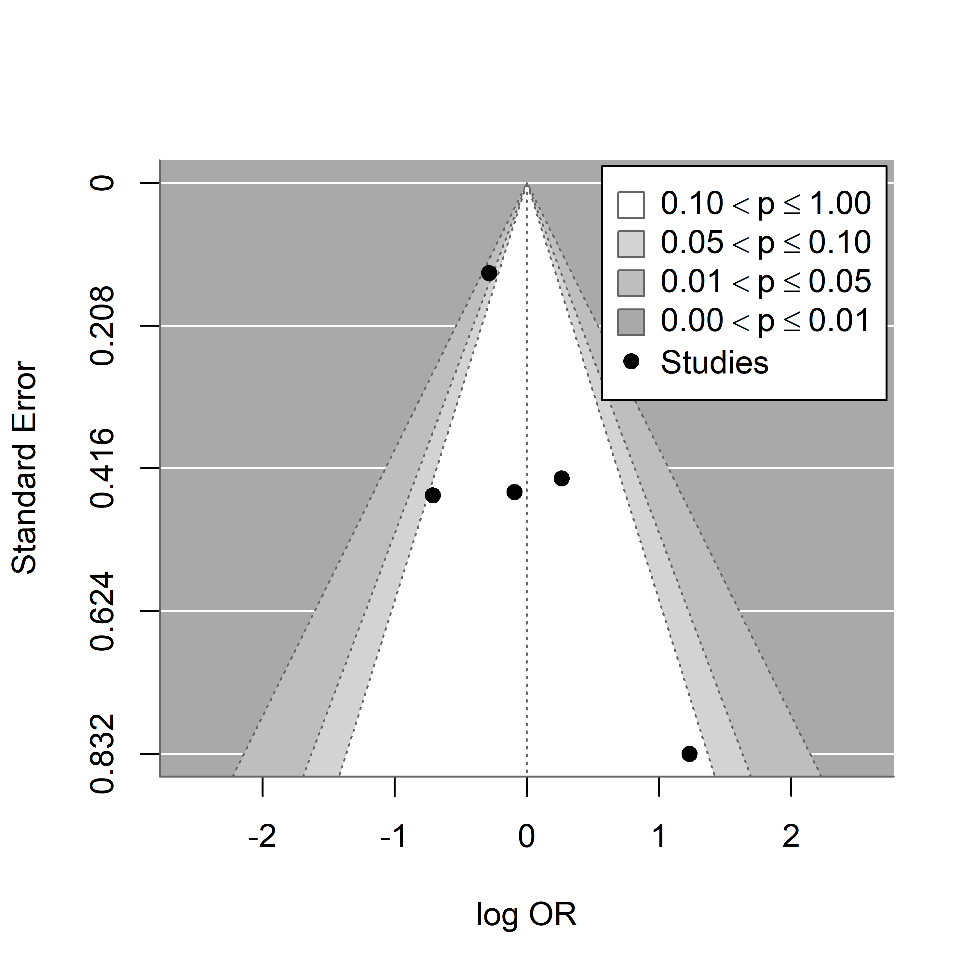


Supplementary Figure 5. Funnel plot for the pooled analysis on alcohol consumption and chemotherapy-induced delayed nausea


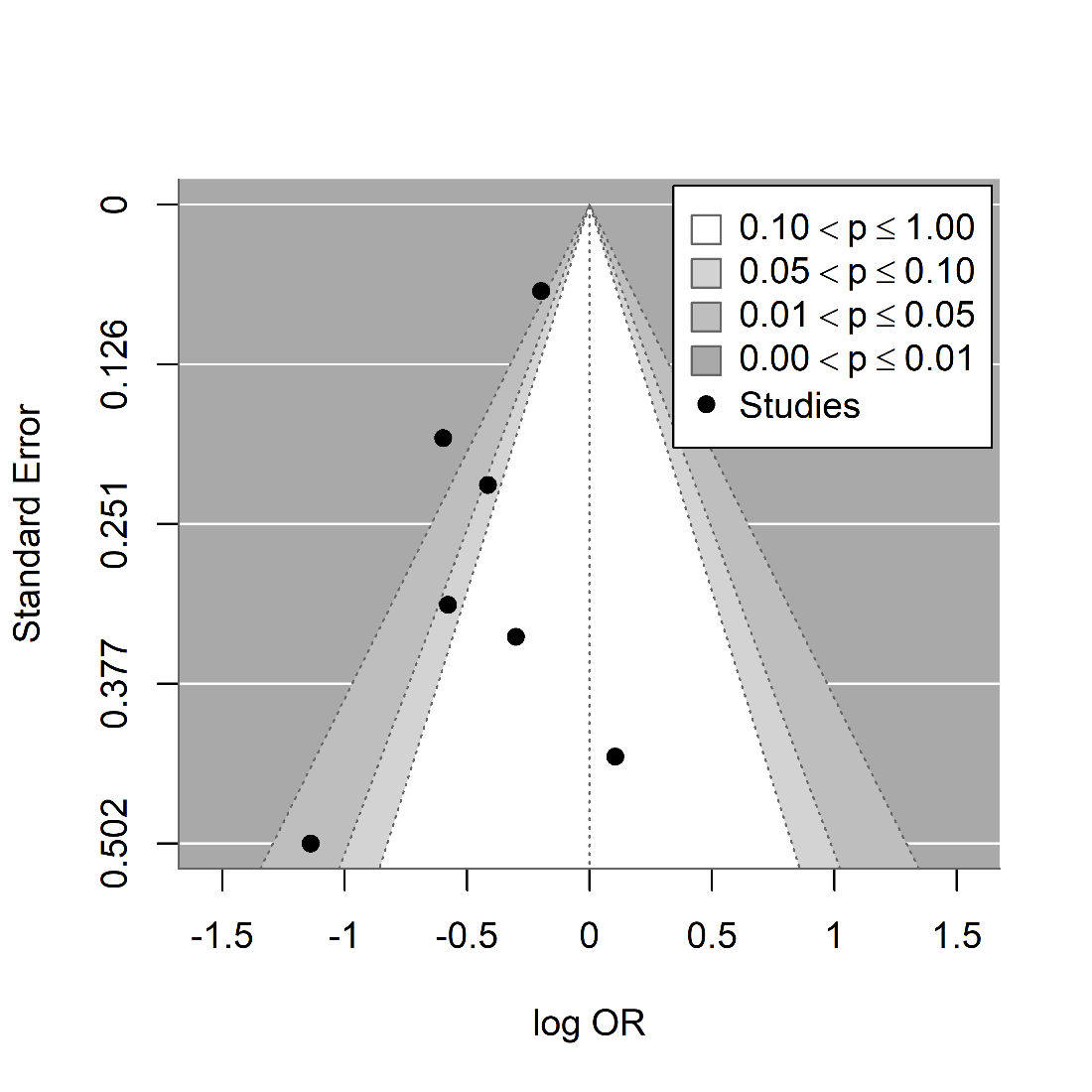


Supplementary Figure 6. Funnel plot for the pooled analysis on alcohol consumption and chemotherapy-induced overall nausea
